# Acute health symptoms related to perception and practice of pesticides use among farmers from all regions of Thailand

**DOI:** 10.3389/fpubh.2023.1296082

**Published:** 2024-01-08

**Authors:** Ratana Sapbamrer, Nalin Sittitoon, Sakesun Thongtip, Eakasit Chaipin, Chatchada Sutalangka, Wilawan Chaiut, Aroon La-up, Phiman Thirarattanasunthon, Ajchamon Thammachai, Boonsita Suwannakul, Noppharath Sangkarit, Amornphat Kitro, Jinjuta Panumasvivat

**Affiliations:** ^1^Department of Community Medicine, Faculty of Medicine, Chiang Mai University, Chiang Mai, Thailand; ^2^School of Environmental Health, Institute of Public Health, Suranaree University of Technology, Nakhon Ratchasima, Thailand; ^3^Department of Environmental Health, School of Public Health, University of Phayao, Phayao, Thailand; ^4^Department of Public Health, Faculty of Science, Rajabhat Lampang University, Lampang, Thailand; ^5^Department of Physical Therapy, School of Integrative Medicine, Mae Fah Luang University, Chiang Rai, Thailand; ^6^Nakhonsawan Campus, Mahidol University, Nakhon Sawan, Thailand; ^7^School of Public Health, Walailak University, Nakhon Si Thammarat, Thailand; ^8^Department of Physical Therapy, School of Allied Health Sciences, University of Phayao, Phayao, Thailand

**Keywords:** pesticide exposure, occupational health, symptom, skin, eye, neurological, neuromuscular, respiratory

## Abstract

**Introduction:**

Occupational exposure to pesticides may cause acute health effects for farmers and agricultural workers. Therefore, this study aims to investigate the prevalence of poisoning symptoms related pesticide exposure among farmers from all regions of Thailand, as well as factors linked to poisoning symptoms of neurological and neuromuscular systems, the respiratory system, and eye and skin disorders.

**Methods:**

A cross sectional study was conducted in 4,035 farmers who lived in four regions of Thailand. The samples were chosen using stratified random sampling, with 746 for the Central region, 2,065 for the North-East, 586 for the North, and 638 for the South.

**Results:**

The results found that the highest prevalence of poisoning symptoms was found in association with neurological and neuromuscular systems (75%), followed by the respiratory system (60.4%), the eyes (41.2%), and skin (14.8%). The most prevalent symptoms were muscle pain (49%) for neurological and neuromuscular symptoms, burning nose (37.6%) for respiratory symptoms, itchy eyes (26.3%) for eye symptoms, and rashes (14.4%) for skin symptoms. The remarkable findings were that types of pesticide use, task on the farm, types of pesticide sprayers, and perception are the crucial factors affecting all poisoning symptoms.

**Discussion:**

The findings are also beneficial to the Thai government and other relevant organizations for launching measures, campaigns, or interventions to lower modifiable risk factors, resulting in reducing health risks associated with pesticide exposure.

## 1 Introduction

The agriculture sector is a major part of the employment demographic in Thailand, including almost one-third of the country’s labor force. A comparable area of employment is in the services sector. Thailand is the largest supplier of rubber, frozen shrimp, canned tuna, and canned pineapple. In 2021, the sectors of agriculture, hunting, and forestry contributed over 39.43 billion US Dollar to the gross domestic product (GDP), of Thailand, and the agriculture sector had the highest contribution. Although the country’s GDP from the agricultural sector is only 6% and GDP growth has been relatively slow, Thailand has been a successful agricultural nation because of the country’s abundant natural resources, which include a range of crops, farms, and fisheries (1, 2).

To enhance crop yield and production, pesticides in agriculture play an important role in controlling pests and weeds worldwide (3). Although there are varieties of methods for increasing crop production, such as organic farming, integrated pest management (IPM), soil management, and other cultivation techniques, pest attack is also a significant factor in crop production losses, resulting in less competition in global markets (4). Oerke (5) mentioned that pre-harvest pests worldwide are responsible for the losses of an average of 35% of the crop yields. In 2020, Thailand imported 98,449 metric tons of agricultural chemicals for a total of 8.9 billion US dollar. The highest quantity of pesticides imported was herbicides (57,007 metric tons), followed by insecticides (18,946 metric tons) and fungicides (15,177 metric tons) (6). Farmers and agricultural workers are a potentially vulnerable population, due to a combination of factors including sociodemographic characteristics, culture, and exposure to pesticides on the farm (7). The majority of studies in Thailand reported urinary dialkyl phosphate (DAP) metabolite levels which is a biomarker of organophosphate exposure. Recent studies in Thailand, farmers in all regions of Thailand had urine DAP levels that ranged from 87.32 to 122.2 microgram/gram creatinine (8–10). Additionally, the study by Suntudrob et al. (11) monitored pesticide residues in vegetables from all provinces of Thailand, and reported that 22.3% of the samples had detectable pesticide residues with a range of <0.01–5.9 mg/kg. According to a systematic review by Boedeker et al. (12), there are 385 million cases of acute pesticide poisoning globally each year, 11,000 of which result in fatalities. Interestingly, 44% of the farmers had poisoning symptoms from exposure to pesticides every year. Occupational exposure to pesticides had been linked with poisoning symptoms including headache, breathlessness, wheeze, nausea, muscle weakness, tremor, eye irritation, and rashes (13–18). In Thailand, the Department of Disease Control at the Ministry of Public Health reported 46,874 cases of pesticide poisoning during 2001–2020 with average of 2,344 cases each year, and 49 of total death cases. In 2020, the Northern region of Thailand had the highest reported number of cases of pesticide poisoning (14.89 cases/100,000 population), followed by the North-East (8.74 cases/100,000 population), Central (7.12 cases/100,000 population), and Southern regions (3.99 cases/100,000 population) (19). The symptoms of pesticide poisoning listed included headache, dizziness, nausea, muscle pain, breathlessness, blurred vison, burning eyes or skin, and others (19). However, the cases reported by the Department of Disease Control were less common than the actual cases. The symptoms of pesticide poisoning are notoriously non-specific, and in many cases health care services did not enter the code for pesticide poisoning (designated as code T60) in the International Classification of Diseases and Related Health Problems 10th Revision (ICD-10). Additionally, farmers with mild symptoms may decide against seeking medical treatment (20). Therefore, the present study aims to investigate the true prevalence of poisoning symptoms related pesticide exposure among farmers from all regions of Thailand, as well as factors linked to problems with the neurological and neuromuscular system, the respiratory system, and eye and skin disorders. The findings from this study reveal both unmodifiable and modifiable risk factors affecting the poisoning symptoms. The findings are also beneficial to Thai government and other relevant organizations for launching measures, campaigns, or interventions to lower modifiable risk factors, resulting in reducing health risks for pesticide exposure.

## 2 Methods

### 2.1 Setting and participants

**Figure 1:**
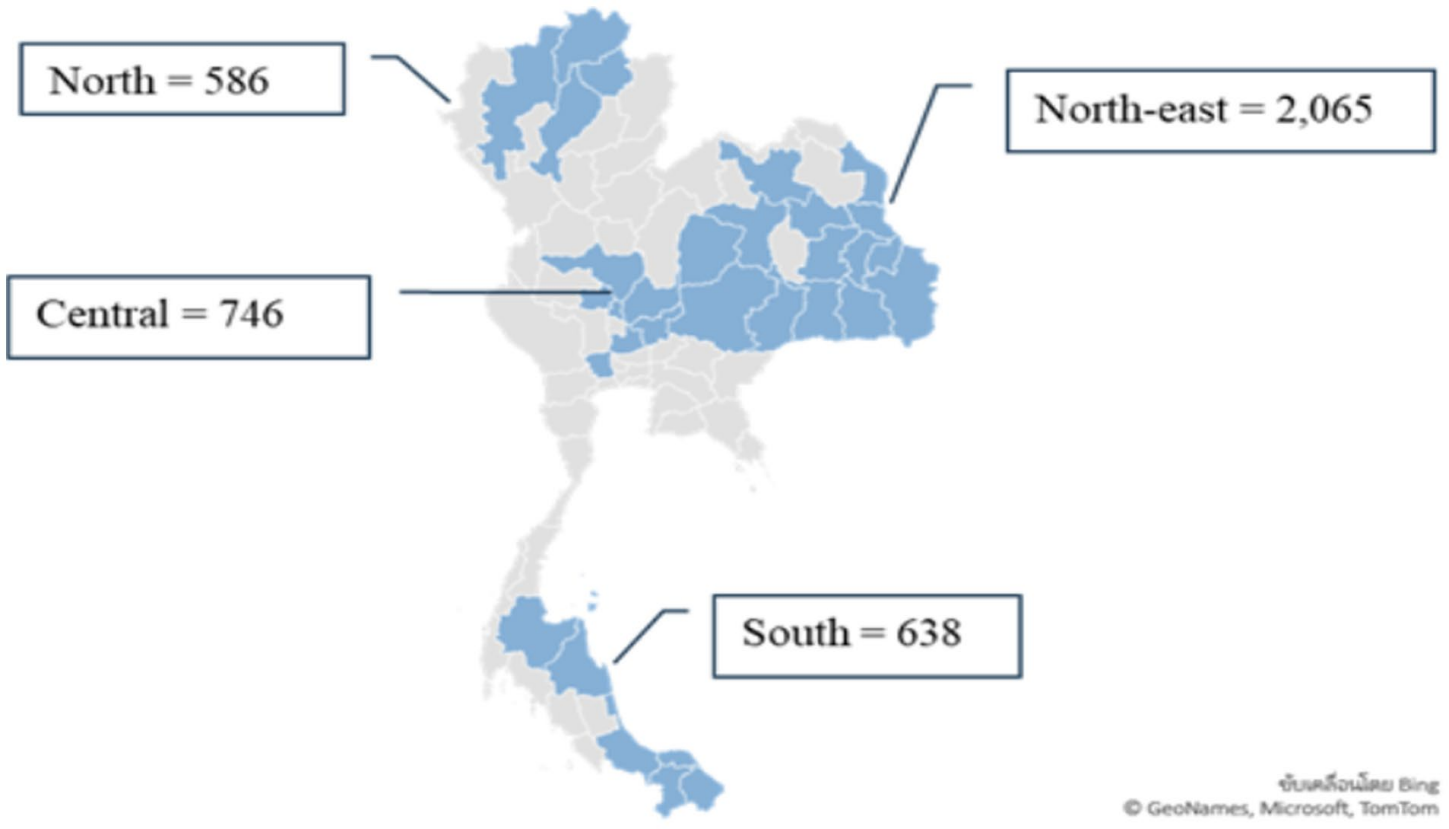
The numbers and provinces of study participants.

A cross sectional study was conducted between January and July 2023. Healthy farmers aged ≥ 18 years who lived in any of the four regions of Thailand and used pesticides for agriculture were invited to participate in the study. The total of the agricultural population in the 4 regions of Thailand was 6,744,856, which were categorized as 1,249,490 in the Central area, 3,503,763 from the North-East, 864,400 from the North, and 1,127,203 from the South (21). The sample size was calculated using the EpiInfo program. The expected frequency of 40%, a confidence limit of 2% and a confidence interval level of 99% were used for calculation. The minimum sample size for representativeness from the calculation was 3,979, and the sample size that was actually collected was 4,035. The samples were chosen using stratified random sampling, resulting in 746 for the Central region, 2,065 for the North-East, 586 for the North, and 638 for the South. Sing Buri, Chai Nat, Ayutthaya, Saraburi, Nakhon Prathom, Lopburi, Nakhon Sawan provinces were chosen as representative areas of the Central region; Khon Kaen, Nakhon Phanom, Chaiyaphum, Yasothon, Roi Et, Amnat Charoen, Kalasin, Mukdahan, Udon Thani, Nakhon Ratchasima, Buriram, Surin, Sisaket, and Ubon Ratchathani provinces as representative of the North-East; Chiang Rai, Chiang Mai, Phayao, and Lam Pang provinces as representative areas of the North and Nakhon Si Thammarat, Narathiwat, Songkhla, Surat Thani, Yala, and Pattani provinces as representative areas of the South. These 31 provinces are all agricultural areas in Thailand (Figure 1).

### 2.2 Interview

Structured and in-person interview was employed as a research instrument. Since Thailand has regional languages and for convenience of traveling for interviews, 30 interviewers (one in each province) were selected based on their home regions. The interviewers were trained by the research team before the participants were interviewed. The topics included in the training of the interviewers included the aim of the study, structure and questions in the questionnaire, and coaching to ensure good performance of the interviewers. Before the interview, the farmers who met the inclusion criteria were invited to participate and were requested to sign a written consent form. The interview took about 10 to 15 min.

The questions in interview form were divided into 4 sections, specifically socio-demographic characteristics, agricultural information, perception and practice regarding safe pesticide use, and poisoning symptoms related to pesticides use. Socio-demographic data included age, gender, marital status, education level, co-morbidity, smoking status, alcohol consumption, and monthly income. Agricultural information investigated was planting status, distance from farm to home, task on the farm, pesticide use, type of pesticide sprayer, and agricultural area. Regarding the perception and practice of safe pesticide use, there were 35 questions for the perception and 35 questions for the practice. The perception questions were measured using a 2-point scale, including yes (1) and no (0). The total summative score ranged from 0 to 35. The practice questions were measured using a 5-point scale, including never (0), seldom (1), sometimes (2), often (3), always (4). The total summative score ranged from 0 to 140.

The poisoning symptoms after exposure to pesticides in the past 3 months were categorized as neurological and neuromuscular, respiratory, eye and skin symptoms. The neurological and neuromuscular symptoms included headache, dizziness, fatigue, fainting, sweating, blurred vision, tremor, cramp, salivating, unconsciousness, convulsion, muscle pain, numbness, and muscle weakness. The respiratory symptoms included burning nose, nasal passages feeling dry, sore throat, asthma, breathlessness, chest tightness, runny nose, cough, and phlegm. The eye symptoms included itchy eyes, burning eyes, and conjunctivitis. The skin symptoms included rashes, red/white pimples, and wounds. Farmers were considered to have poisoning symptoms of those systems if they had at least one symptom from each system. The validity and reliability of the questionnaire were examined prior to data collection. Each questionnaire question received an index of congruence (IOC) score of >0.5, and the overall reliability coefficient for all the questions was 0.77.

### 2.3 Statistical analysis

Descriptive statistics, including frequency (*n*), percentage (%), mean, median, standard deviation (SD.), and percentiles were used. Independent t-test was used to compare the perception and practice regarding safe pesticide use between the farmer with co-morbidity and without co-morbidity. Simple logistic regression, was used to investigate the variables associated with the poisoning symptoms, and crude odds ratio (COR) and 95% confidence intervals (95%CI) were presented. The variables that had a significant (*p*-value < 0.05) from the simple logistic regression were included in the model of multivariable logistic regression. Multivariable logistic regression were used to investigate the factors associated with poisoning symptoms, and adjusted odds ratio (adj.OR) and 95%CI were presented.

## 3 Results

### 3.1 Socio-demographic characteristics and agricultural information

The average age of the farmers was 52.5 ± 10.8 years, and had a median monthly income of 228.6 US Dollar. Approximately 54 % of farmers were female, and 63.0% had elementary school or lower levels of education. Almost 30% of the farmers had a co-morbidity, and 31.6% and 19.0%, respectively, drank alcohol and smoked cigarettes.

The majority of the farmers (78.2%) had their own area for agriculture with an average agricultural area of 5.0 ± 6.1 acres. More than half of the farmers (54.4%) worked on their farm 2–4 h/day, and 64% of them had a distance from farm to home of more than 2 kilometers. The farmers’ tasks included harvesting/packing crops (65.9%), mixing pesticides (62.1%), and spraying pesticides (61.6%). The highest level of pesticide use involved herbicides (77.3%), followed by insecticides (68.8%), and fungicides (54.5%; Table 1).

**Table 1:** Socio-demographic characteristics and agricultural information among Thai farmers (*N* = 4,035).

Parameters		*n* (%) or mean ± SD
**Socio-demographic characteristics**		
Age (yrs.old), mean ± SD.		52.5 ± 10.8
Monthly income (US Dollar), median (P25th–P75th)		228.6 (128.6–285.7)
Gender, *n* (%)	Male	1,841 (45.6)
	Female	2,194 (54.4)
Marital status, *n* (%)	Single/divorced/widowed	920 (22.8)
	Married	3,115 (77.2)
Education, *n* (%)	Elementary school or lower	2,542 (63.0)
	Secondary school or higher	1,493 (37.0)
Co-morbidity, *n* (%)		1,194 (29.6)
		2,841 (70.4)
Smoking status, *n* (%)		767 (19.0)
		3,268 (81.0)
Alcohol consumption, *n* (%)		1,275 (31.6)
		2,760 (68.4)
**Agricultural information**		
Planting status, *n* (%)	Own area	3,157 (78.2)
	Hiring	878 (21.8)
Distance from farm to home, *n* (%)	0–2 km	1,454 (36.0)
	>2 km	2,581 (64.0)
Working hour on farm, *n* (%)	2–4 h./day	2,196 (54.4)
	>4 h./day	1,839 (45.6)
Task on farm, *n* (%)	Mixing pesticide	2,505 (62.1)
	Spraying pesticides	2,486 (61.6)
	Harvesting/Packing	2,660 (65.9)
Pesticides use, *n* (%)	Herbicides	3,120 (77.3)
	Insecticides	2,775 (68.8)
	Fungicides	2,198 (54.5)
	Rodenticides	1,471 (36.5)
	Nematocides	1,986 (49.2)
	Molluscicides	1,580 (39.2)
Types of pesticide sprayer, *n* (%)	Hand knapsack sprayer	1,907 (47.3)
	Motorized knapsack sprayer	2,128 (52.7)
Agricultural area (acres), mean ± SD.		5.0 ± 6.1

### 3.2 Perception and practices regarding safe pesticide use

The most correct statements of farmers’ perceptions regarding the safe use of pesticides were as follows: choice of pesticide based on the types of pests (97.9%); thorough reading of the label on the pesticide before application (97.2%); choice of pesticide in relation to proper labeling and warning (97.0%); a mask should be used while spraying the pesticides (95.3%); and mixing of pesticides in accordance with the label’s instructions (94.5%). However, the least correct statements of farmers’ perceptions regarding the safe use of pesticides were as follows: take a shower immediately after applying pesticides is necessary (13.0%); pesticides can cause air pollution (13.5%); do not eat or drink while mixing pesticides (13.5%); personal protectivie equipment (PPE) should always be worn despite using small amounts of pesticides (16.0%); clothes worn during application of pesticides should be washed before wearing them the next time (17.7%; Table 2). When comparing the perception between the farmer with co-morbidity and without co-morbidity by using independent t-test, the results found that the farmers with co-morbidity had significantly higher perceptions scores (20.0 ± 5.1) than the farmer without co-morbidity (18.9 ± 4.6; *p*-value < 0.001).

**Table 2:** Perception regarding safe pesticide use among Thai farmers (*N* = 4,035).

Questions	Answer correct, *n* (%)
1.Choose pesticides based on the types of pests.	3,952 (97.9)^a^
2.Thoroughly read label on the pesticides before applying pesticides.	3,922 (97.2)^a^
3.Choose pesticides having proper labeling and warning.	3,912 (97.0)^a^
4.Mask should be used while spraying pesticides.	3,847 (95.3)^a^
5.Mix pesticides in accordance with the label’s instruction.	3,813 (94.5)^a^
6.Immediately rinse eyes with water following splashing of pesticide into eyes.	3,756 (93.1)
7.Do not eat or drink while spraying pesticides.	3,634 (90.1)
8.Pesticides can increase risks of cancers.	3,631 (90.0)
9.Pesticides can increase risks of asthma.	3,590 (89.0)
10.Pesticides can cause water pollution.	3,573 (88.6)
11.Applying pesticides during the cooler part of the day.	3,334 (82.6)
12.Pesticides can be flammable.	3,299 (81.8)
13.Upwind pesticide applications are safer than downwind ones.	3,181 (78.8)
14.Empty pesticide containers should be disposed of by burying.	3,170 (78.6)
15.PPE can reduce pesticide exposure.^c^	2,887 (71.5)
16.Pesticides must to be kept in a safe area.	2,779 (68.9)
17.Do not smoke during application of pesticides.	2,615 (64.8)
18.Pesticides are unnecessary for increasing productivity.	2,280 (56.5)
19.There’s no need to use high amounts of pesticides for controlling pests.	1,982 (49.1)
20.Pesticide use is unnecessary for killing pests.	1,877 (46.5)
21.Pesticides can enter to the body through injection, inhalation, and dermal contact.	1,199 (29.7)
22.Do not keep the remaining pesticides that have been mixed for use later	1,129 (28.0)
23.Long-sleeved shirt and trousers alone are not enough to protect against pesticide exposure.	1,100 (27.3)
24.Most pesticides can penetrate though intact skin.	992 (24.6)
25.Empty pesticide containers cannot be reused.	919 (22.8)
26.High prices of pesticides are unnecessary.	912 (22.6)
27.Spray apparatus should not be cleaned in rivers or waterways.	898 (22.3)
28.Pesticides have an effect on animal and pet health	880 (21.8)
29. Masks should be used while mixing pesticides.	775 (19.2)
30.PPE is easy and practical to use.^c^	768 (19.0)
31.Pesticide-applying clothes should be washed before wearing them the next time.	714 (17.7)^b^
32.PPE should always be worn despite using small amount of pesticides.^c^	644 (16.0)^b^
33.Do not eat or drink while mixing pesticides.	544 (13.5)^b^
34.Pesticides can cause air pollution.	544 (13.5)^b^
35.Take a shower immediately after applying pesticides is necessary.	525 (13.0)^b^
Mean ± SD.of perception scores = 19.2 ± 4.8	

^a^The top 5 questions ranked as the highest frequency; ^b^The bottom 5 questions ranked as the lowest frequency; ^c^PPE is defined as PPE, which includes long-sleeved shirt, long-sleeved trousers, hat, mask, boots, goggles, and rubber apron that the Department of Disease Control, Ministry of Public Health, Thailand recommend farmers to wear while applying pesticides to their crops.

The most farmers’ practices regarding the safe use of pesticides were as follows: do not blow through mouth when nozzle is blocked (3.72 ± 0.78); do not take children to the farm during application of pesticides (3.64 ± 0.91); wear long-sleeved trousers while applying pesticides (3.56 ± 0.88); take a shower immediately after applying pesticides (3.52 ± 0.93); and wear boots while applying pesticides (3.52 ± 0.92). The least farmers’ practices regarding the safe use of pesticides were as follows: wear rubber apron while applying pesticides (2.73 ± 1.48); do not keep remaining pesticides that have been mixed for use later (2.67 ± 1.43); do not mix pesticides in a cocktail by considering only more convenience (2.71 ± 1.46); choose less toxic pesticides for killing pests (2.91 ± 1.18); and wear goggles while applying pesticides (3.07 ± 1.26; Table 3). When comparing the practices between the farmer with co-morbidity and without co-morbidity by using independent t-test, the results found that no differences in practice scores between the farmers with and without co-morbidity (114.5 ± 21.9 for farmers with co-morbidity and 115.4 ± 21.6 for farmers without co-morbidity; *p*-value = 0.229).

**Table 3:** Practices regarding safe pesticide use among Thai farmers (*N* = 4,035).

Questions	Scores (mean ± SD)	*n* (%)				
		Never	Seldom	Sometimes	Often	Always
1.When nozzle blocking, do not blow it through mouth.	3.72 ± 0.78 ^a^	55 (1.4)	90 (2.2)	226 (5.6)	174 (4.3)	3,490 (86.5)
2.Do not take children to farm during application of pesticides.	3.64 ± 0.91^a^	104 (2.6)	114 (2.8)	230 (5.7)	240 (5.9)	3,347 (82.9)
3. Wear long-sleeved trousers while applying pesticides.	3.56 ± 0.88^a^	104 (2.6)	91 (2.3)	174 (4.3)	744 (18.4)	2,922 (72.4)
4.Take a shower immediately after applying pesticides.	3.52 ± 0.93 ^a^	112 (2.8)	98 (2.4)	258 (6.4)	689 (17.1)	2,878 (71.3)
5. Wear boots while applying pesticides.	3.52 ± 0.92^a^	103 (2.6)	115 (2.9)	224 (5.6)	741 (18.4)	2,852 (70.7)
6.Do not use hands for rubbing eyes or scratching skin.	3.50 ± 0.94	80 (2.0)	143 (3.5)	368 (9.1)	526 (13.0)	2,918 (72.3)
7.Wear long-sleeved shirts while applying pesticides.	3.49 ± 0.98	163 (4.0)	93 (2.3)	181 (4.5)	761 (18.9)	2,837 (70.3)
8.Keep pesticides away from children and pets.	3.48 ± 1.04	204 (5.1)	93 (2.3)	175 (4.3)	642 (15.9)	2,921 (72.4)
9.Wear masks while applying pesticides.	3.46 ± 0.97	117 (2.9)	149 (3.7)	262 (6.5)	748 (18.5)	2,759 (68.4)
10.Wash the hair immediately after applying pesticides.	3.45 ± 0.98	130 (3.2)	110 (2.7)	329 (8.2)	726 (18.0)	2,740 (67.9)
11.Change pesticide-applying clothes before eating or drinking.	3.45 ± 0.98	73 (1.8)	204 (5.1)	398 (9.9)	522 (12.9)	2,838 (70.3)
12.Do not use mouth for opening/tearing pesticide bottle/pack.	3.43 ± 1.23	289 (7.2)	204 (5.1)	198 (4.9)	153 (3.8)	3,191 (79.1)
13.Read instruction thoroughly before applying pesticides	3.42 ± 0.99	140 (3.5)	119 (2.9)	281 (7.0)	873 (21.6)	2,622 (65.0)
14.Wear hat while applying pesticides.	3.39 ± 1.00	135 (3.3)	125 (3.1)	370 (9.2)	826 (20.5)	2,579 (63.9)
15.Change clothes immediately after applying pesticides.	3.37 ± 1.09	218 (5.4)	98 (2.4)	347 (8.6)	686 (17.0)	2,686 (66.6)
16.Wear gloves while applying pesticides.	3.37 ± 1.06	138 (3.4)	226 (5.6)	300 (7.4)	706 (17.5)	2,665 (66.0)
17.Survey types of pests before buying pesticides.	3.36 ± 0.96	99 (2.5)	152 (3.8)	351 (8.7)	1,045 (25.9)	2,388 (59.2)
18.Choose types and quantities of pesticides based on types of pests.	3.36 ± 0.98	114 (2.8)	149 (3.7)	348 (8.6)	976 (24.2)	2,448 (60.7)
19.Choose pesticides having proper labeling and warning.	3.34 ± 1.01	105 (2.6)	215 (5.3)	322 (8.0)	959 (23.8)	2,434 (60.3)
20.Do not smoking, drinking, or eating during applying pesticides.	3.29 ± 1.17	201 (5.0)	251 (6.2)	377 (9.3)	542 (13.4)	2,664 (66.0)
21.Mix pesticides in accordance with the label’s instructions.	3.27 ± 1.04	152 (3.8)	165 (4.1)	370 (9.2)	1,105 (27.4)	2,243 (55.6)
22.Clean spraying equipment before storage.	3.27 ± 1.19	274 (6.8)	170 (4.2)	310 (7.7)	716 (17.7)	2,565 (63.6)
23.Mix pesticides outdoors	3.22 ± 1.08	172 (4.3)	193 (4.8)	388 (9.6)	1,101 (27.3)	2,181 (54.1)
24.Do not use hands without gloves for mixing pesticides.	3.20 ± 1.26	267 (6.6)	306 (7.6)	383 (9.5)	485 (12.0)	2,594 (64.3)
25.Stop spraying pesticides while windy.	3.19 ± 1.20	224 (5.6)	274 (6.8)	436 (10.8)	689 (17.1)	2,412 (59.8)
26.Mix pesticides upwind.	3.17 ± 1.18	261 (6.5)	193 (4.8)	401 (9.9)	930 (23.0)	2,250 (55.8)
27.Do not make a phone call during application of pesticides.	3.17 ± 1.11	143 (3.5)	257 (6.4)	552 (13.7)	887 (22.0)	2,196 (54.4)
28. Separately wash pesticide-applying clothes from other clothes.	3.16 ± 1.37	399 (9.9)	278 (6.9)	303 (7.5)	355 (8.8)	2,700 (66.9)
29.Check spraying equipment before spraying pesticides.	3.15 ± 1.21	301 (7.5)	164 (4.1)	378 (9.4)	966 (23.9)	2,226 (55.2)
30.Spray pesticides upwind.	3.14 ± 1.22	325 (8.1)	144 (3.6)	401 (9.9)	952 (23.6)	2,213 (54.8)
31.Wear goggles while applying pesticides.	3.07 ± 1.26 ^b^	274 (6.8)	320 (7.9)	477 (11.8)	761 (18.9)	2,203 (54.6)
32.Choose less toxic pesticides for killing pests.	2.91 ± 1.18 ^b^	196 (4.9)	366 (9.1)	752 (18.6)	1,016 (25.2)	1,705 (42.3)
33.Do not mix pesticides in a cocktail by considering only more convenience.	2.71 ± 1.46 ^b^	495 (12.3)	516 (12.8)	568 (14.1)	547 (13.6)	1,909 (47.3)
34.Do not keep remaining pesticides that have been mixed for use later.	2.67 ± 1.43 ^b^	433 (10.7)	578 (14.3)	679 (16.8)	558 (13.8)	1,787 (44.3)
35.Wear a rubber apron while applying pesticides.	2.73 ± 1.48 ^b^	555 (13.8)	422 (10.5)	494 (12.2)	641 (15.9)	1,923 (47.7)
Total	115.1 ± 21.7					

^a^The top 5 questions ranked the highest score; ^b^The bottom 5 questions ranked the lowest score.

### 3.3 Poisoning symptoms related to pesticide exposure among Thai farmers

**Figure 2:**
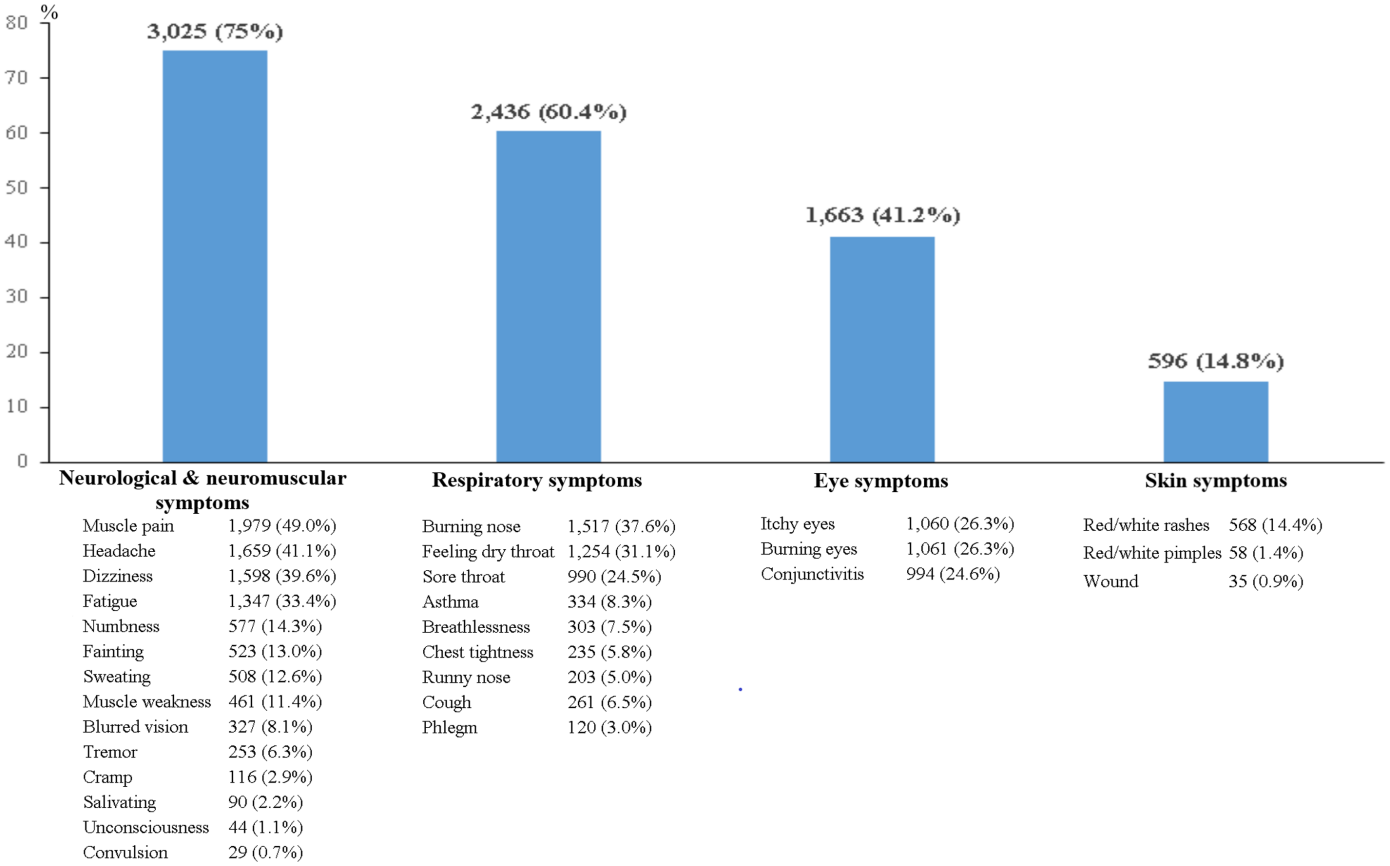
Poisoning symptoms related to pesticide exposure among Thai farmers (*N* = 4,035).

The highest prevalence of poisoning symptom was found to be associated with neurological and neuromuscular symptoms (75%), followed by respiratory symptoms (60.4%), eye symptoms (41.2%), and then skin symptoms (14.8%). Regarding symptoms associated with neurological and neuromuscular systems, the top three symptoms were muscle pain (49%), headache (41.1%), and dizziness (39.6%). The top three respiratory symptoms were burning nose (37.6%), feeling dry throat (31.1%), and sore throat (24.5%). The highest eye and skin symptoms were itchy eyes (26.3%) and rashes (14.4%; Figure 2).

### 3.4 Factors associated with poisoning symptoms among Thai farmers

Factors associated with poisoning symptoms among Thai farmers after adjustment for socio-demographic characteristics are presented in Table 4.

**Table 4:** Factors associated with poisoning symptoms among Thai farmers (*N* = 4,035).

Factors	Neurological symptoms		Respiratory symptoms		Eye symptoms		Skin symptoms	
	COR (95%CI)	Adj.OR (95%CI)	COR (95%CI)	Adj.OR (95%CI)	COR (95%CI)	Adj.OR (95%CI)	COR (95%CI)	Adj.OR (95%CI)
**Socio-demographic factors**								
Age	1.00(0.99, 1.01)	0.99(0.99, 1.01)	1.01(1.00, 1.01)^**^	1.00(0.99, 1.01)	1.02(1.01, 1.02)^**^	1.02(1.01, 1.02)^**^	1.00(0.99, 1.01)	1.00(0.99, 1.01)
Gender	1.49(1.29, 1.72)^**^	1.20(0.99, 1.44)	1.17(1.03, 1.32)^*^	0.88(0.75, 1.04)	1.23(1.08, 1.39)^**^	1.17(0.99, 1.37)	1.19(1.00, 1.42)^*^	1.04(0.83, 1.30)
Marital status	1.12(0.95, 1.34)	1.15(0.96, 1.38)	1.02(0.88, 1.19)	1.05(0.89, 1.24)	1.04(0.89, 1.21)	1.09(0.93, 1.29)	0.65(0.52, 0.82)^**^	0.64(0.51, 0.82)^**^
Education level	1.39(1.20, 1.61)^**^	1.23(1.04, 1.46)^*^	1.13(0.99, 1.29)	0.94(0.80, 1.10)	0.81(0.71, 0.92)^**^	0.55(0.47, 0.64)^**^	0.96(0.80, 1.14)	0.92(0.74, 1.15)
Smoking status	1.30(1.07, 1.57)^**^	0.94(0.74, 1.18)	1.32(1.12, 1.55)^**^	1.33(1.08, 1.63)^**^	1.13(0.97, 1.33)	1.25(1.02, 1.53)^*^	1.08(0.86, 1.14)	1.14(0.86, 1.15)
Alcohol consumption	1.33(1.13, 1.56)^**^	1.09(0.89, 1.32)	1.16(1.01, 1.33)^*^	1.02(0.86, 1.22)	0.86(0.75, 0.98)^*^	0.74(0.62, 0.88)^**^	0.84(0.70, 1.02)	0.85(0.67, 1.09)
Co-morbidity	0.86(0.74, 1.00)	0.9(0.77,1.08)	1.12(0.97, 1.28)	1.34(1.14, 1.57)^**^	1.16(1.01, 1.33)^*^	1.36(1.16, 1.59)^**^	0.55(0.44, 0.68)^**^	0.72(0.57, 0.91)^**^
**Agricultural factors**								
Planting status	1.37(1.14 1.65)^**^	1.24(1.01, 1.53)^*^	1.24(1.06, 1.45)^**^	1.07(0.89, 1.28)	1.27(1.09, 1.48)^**^	1.47(1.23, 1.76)^**^	0.41(0.32, 0.54)^**^	0.53(0.39, 0.72)^**^
Distance from farm to home	1.36(1.17, 1.57)^**^	1.51(1.28, 1.77)^**^	1.45(1.27, 1.65)^**^	1.54(1.33, 1.79)^**^	1.30(1.14, 1.49)^**^	1.38(1.18, 1.60)^**^	1.71(1.41, 2.08)^**^	1.21(0.97, 1.51)
Working hours on farm	1.46(1.26, 1.68)^**^	1.43(1.21, 1.69)^**^	1.42(1.25, 1.62)^**^	1.51(1.29, 1.75)^**^	1.07(0.94, 1.21)	1.07(0.92, 1.24)	0.70(0.58, 0.83)^**^	0.96(0.77, 1.18)
Mixing pesticides	2.21(1.91, 2.56)^**^	1.67(1.31, 2.11)^**^	1.75(1.54, 1.99)^**^	1.49(1.19, 1.87)^**^	1.41(1.24, 1.61)^**^	0.80(0.63, 1.00)	1.62(1.34, 1.96)^**^	1.45(1.05, 1.98)^*^
Spraying pesticides	1.95(1.69, 2.25)^**^	0.96(0.75, 1.23)	1.55(1.54, 1.99)^**^	0.89(0.71, 1.11)	1.57(1.38, 1.79)^**^	1.18(0.94, 1.49)	1.18(0.99, 1.42)	0.57(0.42, 0.78)^**^
Harvesting/packing	1.64(1.42, 1.90)^**^	1.12(0.95, 1.33)	2.03(1.78, 2.32)^**^	1.53(1.32, 1.78)^**^	1.85(1.62, 2.13)^**^	1.27(1.09, 1.49)^**^	2.23(1.80, 2.75)^**^	1.76(1.38, 2.25)^**^
Herbicides used	1.91(1.63, 2.24)^**^	0.92(0.73, 1.16)	1.62(1.40, 1.88)^**^	1.04(0.84, 1.30)	1.95(1.67, 2.29)^**^	0.92(0.72, 1.16)	1.54(1.23, 1.94)^**^	0.96(0.66, 1.38)
Insecticides used	2.02(1.74 2.34)^**^	1.19(0.95, 1.48)	1.65(1.44, 1.89)^**^	0.99(0.81, 1.22)	2.65(2.29, 3.07)^**^	1.89(1.52, 2.35)^**^	1.77(1.44, 2.18)^**^	0.88(0.63, 1.24)
Fungicides used	2.03(1.75, 2.34)^**^	1.25(1.01, 1.54)^*^	1.64(1.44, 1.86)^**^	1.02(0.84, 1.24)	2.24(1.97, 2.55)^**^	1.35(1.11, 1.64)^**^	2.65(2.18, 3.21)^**^	2.03(1.51, 2.73)^**^
Rodenticides used	1.86(1.59, 2.18)^**^	1.04(0.83, 1.31)	2.03(1.77, 2.37)^**^	1.73(1.42, 2.12)^**^	2.30(2.02, 2.62)^**^	1.53(1.26, 1.87)^**^	2.58(2.16, 3.08)^**^	1.47(1.11, 1.94)^**^
Nematocides used	1.85(1.60, 2.14)^**^	1.16(0.94, 1.44)	1.47(1.30, 1.67)^**^	0.85(0.70, 1.03)	1.95(1.72, 2.21)^**^	0.98(0.81, 1.18)	1.77(1.48, 2.12)^**^	0.76(0.58, 1.00)
Molluscicides used	1.92(1.64, 2.24)^**^	1.19(0.97, 1.46)	2.09(1.83, 2.39)^**^	1.17(0.97, 1.40)	1.93(1.69, 2.19)^**^	0.94(0.78, 1.12)	2.71(2.27, 3.23)^**^	1.73(1.33, 2.26)^**^
Types of sprayers	1.40(1.21, 1.61)^**^	1.49(1.28, 1.75)^**^	1.46(1.28, 1.65)^**^	1.62(1.40, 1.87)^**^	1.90(1.67, 2.15)^**^	2.02(1.75, 2.34)^**^	2.17(1.81, 2.59)^**^	1.69(1.37, 2.07)^**^
**Perception and practice factors**								
Perception scores	0.97(0.95, 0.98)^**^	0.97(0.95, 0.98)^**^	0.91 (0.89, 0.92)^**^	0.90(0.89, 0.92)^**^	0.96(0.95, 0.97)^**^	0.96(0.95, 0.98)^**^	0.89(0.87, 0.91)^**^	0.90(0.88, 0.92)^**^
Practices scores	1.00(0.99, 1.00)	0.996(0.992, 1.0)	1.00 (1.00, 1.01)	1.00(0.99, 1.00)	1.01(1.01, 1.02)	1.01(1.00, 1.01)	0.99(0.99, 0.99)	0.98(0.98, 0.99)^**^
Constant		2.30^*^		1.961^*^		0.05^**^		1.59

^*^p-value < 0.05; ^**^p-value < 0.01; Adj.OR, adjusted odds ratio; COR, cruded odds ratio; 95%CI, 95% confidence interval; ref., reference. Defining categorical variables: gender [male vs. female (ref.)]; marital status [single/divorced/widowed vs. married (ref.)]; education level [elementary school or lower vs. secondary school or higher (ref.)]; smoking status [yes vs. no (ref.)]; alcohol consumption [yes vs. no (ref.)]; co-morbidity [yes vs. no (ref.)]; planting status [own farm vs. hiring (ref.)]; distance from farm to home [0–2 km vs. >2 km (ref.)]; working hours on the farm [>4 h./day vs. 2–4 h./day (ref.)]; mixing pesticides [yes vs. no (ref.)]; Spraying pesticides [yes vs. no (ref.)]; harvesting/packing crops [yes vs. no (ref.)]; herbicides use [yes vs. no (ref.)]; insecticides used [yes vs. no (ref.)]; fungicides used [yes vs. no (ref.)]; rodenticides used [yes vs. no (ref.)]; nematocides used [yes vs. no (ref.)]; molluscicides used [yes vs. no (ref.)]; types of sprayers [hand knapsack vs. motorized knapsack (ref.)].

#### 3.4.1 Neurological and neuromuscular symptoms

Factors associated with neurological symptoms included planting on their own farm (*p*-value = 0.041), distance from farm to home 0–2 km (*p*-value < 0.001), working hours on the farm > 4 h./day (*p*-value < 0.001), mixing pesticide tasks (*p*-value < 0.001), fungicides used (*p*-value = 0.041), use of a hand knapsack sprayer (*p*-value < 0.001), and total scores of perception (*p*-value < 0.001).

#### 3.4.2 Respiratory symptoms

Factors associated with the symptoms of respiratory system included distance from farm to home 0–2 (*p*-value < 0.001), working hours on the farm > 4 h/day (*p*-value < 0.001), mixing pesticide task (*p*-value < 0.001), harvesting/packing tasks (*p*-value < 0.001), rodenticides used (*p*-value < 0.001), use of hand knapsack sprayer (*p*-value < 0.001), and total scores of perception (*p*-value < 0.001).

#### 3.4.3 Eye symptoms

Factors associated with eye symptoms included planting on their own farm (*p*-value < 0.001), distance from farm to home 0–2 km (*p*-value < 0.001), harvesting/packing tasks (*p*-value < 0.001), insecticides used (*p*-value < 0.001), fungicides used (*p*-value < 0.001), rodenticides used (*p*-value < 0.001), use of hand knapsack sprayer (*p*-value < 0.001), and total scores of perception (*p*-value < 0.001).

#### 3.4.4 Skin symptoms

Factors associated with skin symptoms included planting on own farm (*p*-value < 0.001), mixing pesticide task (*p*-value = 0.022), spraying pesticide task (*p*-value < 0.001), harvesting/packing tasks (*p*-value < 0.001), fungicides used (*p*-value < 0.001), rodenticides used (*p*-value < 0.001), molluscicides used (*p*-value < 0.001), use of hand knapsack sprayer (*p*-value < 0.001), total scores of perception (*p*-value < 0.001), and total scores of practice (*p*-value < 0.001).

## 4 Discussion

Types of pesticides used in agriculture are the crucial factors affecting poisoning symptoms. According to our findings, farmers who used fungicides on their farms had a higher prevalence of neurological and neuromuscular symptoms. The highest quantities of fungicides imported into Thailand were propineb, mancozeb, and carbendazim (6). *In vivo* and *in vitro* studies suggested that propineb and mancozeb, which were classified in the dithiocarbamate group, may affect the nervous system through inhibition of acetylcholinesterase (AChE) activity, leading to accumulation of acetylcholine at the cholinergic receptors and continuous stimulation of the muscles, glands, and central nervous system. These dithiocarbamate fungicides can also increase the production of ROS and induce oxidative stress (22–25). Mancozeb is polymeric salt of ethylenebisdithiocarbamic acid containing 20%manganese and 2.5% zinc. Exposure to the high doses of manganese contained in mancozeb may result in neurological dysfunction (26). Carbendazim were also affect the nervous system by inhibiting differentiation of neural tissues and inducing oxidative stress (27, 28).

Our findings also found that farmers who used rodenticides on their farms had a higher incidence of respiratory symptoms. The most frequently used rodenticides in Thailand are zinc phosphide and coumarin (6). Zinc phosphide is an inorganic compound that combines zinc with phosphorus. When this compound reacts with moisture in the air, phosphine gas, an active pesticide, is released into the air (29). The phosphine gas can be absorbed systemically and cause the accumulation of fluid in the lungs, resulting in respiratory difficulties (30). Therefore, respiratory failure is the main cause of the death. The phosphine gas can be also react with hydrogen peroxide to form the highly reactive hydroxyl radical that causes lipid peroxidation leading to oxidative damage of cellular membranes and hence cell death (29). It is probable that pathophysiological alterations in the respiratory system may be caused by oxidative stress.

Direct contact of pesticides with the eyes or skin may result in local irritation. Our findings showed that farmers who used insecticides, fungicides, and rodenticides on their farms had a higher prevalence of eye symptoms. The most common symptoms found in this study were itchy eyes (26.3%), followed by burning eyes (26.3%), and conjunctivitis (24.6%). The most common insecticides imported into Thailand during 2019–2020 are abamectin, chlorpyrifos, and carbofuran (6). Abamectin is classified as a serious irritant to eyes and skin (31). Whereas chlorpyrifos is categorized as an organophosphate insecticide, and carbofuran as a carbamate insecticide. Organophosphate and carbamate insecticides have been shown to inhibit AChE activity, resulting in ACh accumulation at synapses and neuromuscular junctions, subsequently causing cholinergic symptoms (32). Animal studies also found that cholinesterase enzymes were detected in ocular tissues related to organophosphate inhibition, including the cornea, choroid, iris, retina, and extraocular muscles (33). Available previous studies mentioned that occupational exposure to pesticides was linked to retinal degeneration in farmers and wives of farmers (34, 35). According to our findings, 26.5% of farmers reported using goggles either never, seldom, or sometimes while applying pesticides. Goggles and rubber apron were the PPE that the least used by the farmers while applying pesticides (54.6 and 47.7%, respectively). It is extremely likely that farmers who did not always wear goggles during pesticide application had a higher risk of being exposed to pesticides via ocular exposure, which may lead to problems with ocular toxicity.

Regarding skin symptoms, our findings showed that farmers who used fungicides, rodenticides, and molluscicides on their farms had a higher prevalence of skin symptoms. Rashes were the most prevalent symptoms found (14.4%). The most common fungicides imported into Thailand are propineb, carbendazim, and mancozeb, while the most common rodenticides are zinc phosphide and coumarin, and the most frequent molluscicide is metaldehyde. All these pesticides are lipophilic, therefore they can pass across the skin cell membrane. Skin contact is its main routes of toxicity for these pesticides, which may result in dermal sensitization and contact dermatitis. Propineb, carbendazim, mancozeb, and coumarin are classified as skin sensitizers which can induce an allergic reaction in the skin, leading to skin redness (26, 36, 37). Zinc phosphide and metaldehyde are classified as irritants to the eyes and skin (38, 39). A study by Corsini et al. (40) mentioned that dithiocarbamate fungicides may activate T cells and natural killer cells, and also induce B cells to secrete more immunoglobulins. The dithiocarbamate fungicides also act as haptens, conjugate to proteins, and may cause allergic hypersensitivity (41). These mechanisms may instigate a systematic autoimmune disorder, resulting in a severe mucosal and cutaneous response (42). In addition, a study by Shin et al. (43) also suggested that dithiocarbamate fungicides can suppress *cutibacterium acne* and induce skin inflammation. Importantly, our results showed that the rubber apron was the PPE that the least used by the farmers while applying pesticides (47.7%), leading to a higher incidence of skin contact. Additionally, most farmers (87%) perceived that taking a shower immediately applying pesticides is unnecessary. These perceptions and practices of farmers could increase the chance of exposure to pesticides, and may result in skin irritation and skin allergy. Therefore, wearing of protective clothing, including long-sleeved shirt, long-sleeved trousers, goggles, gloves, hats, and boots, and use of a rubber apron during application of pesticides could reduce the risks of direct contact.

Tasks on the farm are significant factors of exposure to pesticides and health poisoning. Our findings showed that the task of mixing the pesticide was associated with an increasing prevalence of neurological, neuromuscular, respiratory, and skin symptoms. These results were consistent with previous studies (44–46). Dermal and inhalation exposure are the main routes of pesticide exposure in agricultural workers. According to a review by Garzia et al. (47), the mixing tasks showed a higher risk for dermal exposure than spraying and harvesting tasks. Pesticide exposure during the mixing task often occurs as a single and high dose via spills, splashes, and inhalation. When the high doses of pesticides enter the body, they are absorbed, distributed via the blood stream, and consequently cause acute symptoms (48). Our findings also showed that harvesting/packing tasks were associated with increasing prevalence of respiratory, eye, and skin symptoms. It has been acknowledged that pesticides residues have been detected in Thailand’s vegetable and fruit crops (49). It is possible that skin contact and dermal poisoning might occur when farmers have direct contact with agricultural crops that had pesticide residues.

Regarding type of pesticide sprayers, farmers who used a hand knapsack sprayer had a higher prevalence of neurological and respiratory symptoms as well as eye and skin symptoms, compared with those who used a motorized knapsack sprayer. A review by Garzia et al. (47) suggested that applicators who used a knapsack boom spray had higher dermal exposure than those who used a tractor mounted sprayer. A tractor sprayer with an elevated boom and open cap also resulted in higher dermal exposure than use of a tractor without elevated boom and enclosed cap. However, most Thai farmers had limited budgets for farming, so they often used hand knapsack or motorized knapsack sprayers, and they never had access to use of a tractor sprayer. Our results were consistent with the study by Sidthilaw et al. (50) which found that backpack sprayers were at greater risk of herbicide exposure than motorized pump sprayers. The backpack sprayers held the pesticide tank on their backs, and sprayed the pesticides by hands. Therefore, these farmers were probably exposed to pesticides when they pumped them with their hands while carrying the pesticide tank on their backs. Direct contact with pesticides on the hands and the backs may result in dermal sensitization and dermal irritation. Additionally, farmers could inhale pesticide droplets or contact with eyes that were suspended in the air due to spraying, causing eye and skin irritation as well as other poisoning symptoms (51).

Perception and practice regarding safe pesticide use are also factors associated with poisoning symptoms. Our findings found that farmers with higher perceptive scores had a lower prevalence of all symptoms, and farmers with higher practice scores had a lower prevalence of skin symptoms. Previous studies revealed that lack of knowledge, attitude, and practice (KAP) of pesticide use is a major cause of pesticide misuse and poisoning symptom related to pesticide exposure (13, 52, 53). Additionally, our findings show that the least farmers’practices regarding safe pesticide were as follows: wear rubber apron while applying pesticides; do not keep remaining pesticides that have been mixed for use later; do not mix pesticides in a cocktail; choose less toxic pesticides for killing pests; and wear goggles while applying pesticides. The findings were consistent with a systematic review by Sapbamrer and Thammachai (54), suggesting that the lowest uses of specific PPE among pesticide handlers in all world regions were an apron, goggles, gloves, boots, and mask, respectively. It is possible that farmers did not understand the message on pesticide labels, especially pictograms and color codes. Although the pictograms and color codes on pesticide label are intended to be readily understood by farmers with varying languages and literacy levels, previous studies conducted in several countries showed a varied understanding of the massage on pesticide labels (55, 56). Lack of training is also a major cause of pesticide misuse (53). Therefore, continuous training regarding safe pesticide use by using practical methods is urgently need. A study by Maddah et al. (57) suggested that community-based intervention program significantly enhanced the farmers’ KAP regarding safe pesticide use. Training program for understanding and interpreting pictograms and color codes on pesticide labels are necessary (55, 56). Importantly, government plays a vital role in introducing and educating farmers on alternative pest management, such as integrated pest management (IPM) and organic farming approaches for controlling pests and minimizing risks to humans and the environment (53, 58, 59). Promotion of supportive production inputs, information, technology, markets, and certification system for alternative pest management should be implemented by the government (58, 59).

The data were collected from 31 provinces with stratified random sampling in all regions of Thailand, which represented 40.3% of the total provinces. Therefore, the findings can be accepted as a good representative of these factors in Thai farmers. However, there are some limitations in the study. First, the cross-sectional design could only determine the correlation between pesticide exposure and poisoning symptoms, but not confirm causes and health effects. Second, self-reported interviews were used to assess pesticide exposure and poisoning symptoms, over- or under estimation effects might have occurred. In addition, some poisoning symptoms were rather non-specific, indicating that pesticide exposure may not be the cause of the poisoning symptoms occurring. Third, we were unable collect the common names or active ingredients of the pesticides used in farming due to low literacy levels among the farmers. Finally, recall bias might be occurred because the subjects may well have forgotten poisoning symptoms related to pesticide use in the past 3 months. These limitations should be considered during the course of further investigations.

## 5 Conclusion

Neurological and neuromuscular symptoms had the highest prevalence of association with pesticide use and were followed by respiratory symptoms, eye symptoms, and skin symptoms. Types of pesticide use, tasks on the farm, and types of pesticide sprayers are the crucial factors affecting poisoning symptoms related to pesticide exposure. Perceptions regarding safe pesticide use are also factors associated with poisoni symptoms. Therefore, a life-long education program with continual training by using practical methods to change the perceptions and practices of pesticide handlers is urgently needed. Training program for understanding pictograms and color codes on pesticide labels are necessary. Training program on alternative pest management, such as IPM and organic farming, is also necessary to provide options and strategies in controlling pests. Importantly, the establishment of promotion, strategies, legislations, and policies by the government is a key driver for reducing pesticide use sustainably. Promotion of supportive production inputs, information, technology, markets, and certification system for alternative pest management is also needed for solving pest problem and minimizing risks to humans and the environment sustainably.

## Data availability statement

The original contributions presented in the study are included in the article/supplementary material, further inquiries can be directed to the corresponding author.

## Ethics statement

The studies involving humans were approved by the Research Ethics Committee of the Faculty of Medicine, Chiang Mai University, Thailand (No. 419/2022). The studies were conducted in accordance with the local legislation and institutional requirements. The participants provided their written informed consent to participate in this study.

## Author contributions

RS: Conceptualization, Data curation, Formal analysis, Funding acquisition, Investigation, Methodology, Project administration, Resources, Software, Supervision, Validation, Visualization, Writing – original draft, Writing – review & editing. NSi: Conceptualization, Data curation, Formal analysis, Investigation, Methodology, Writing – review & editing. ST: Data curation, Formal analysis, Investigation, Methodology, Writing – review & editing. EC: Data curation, Formal analysis, Investigation, Methodology, Writing – review & editing. CS: Data curation, Formal analysis, Investigation, Methodology, Writing – review & editing. WC: Investigation, Writing – review & editing. AL-u: Data curation, Formal analysis, Investigation, Methodology, Writing – review & editing. PT: Data curation, Formal analysis, Investigation, Methodology, Writing – review & editing. AT: Formal analysis, Writing – review & editing. BS: Formal analysis, Writing – review & editing. NSa: Formal analysis, Writing – review & editing. AK: Formal analysis, Writing – review & editing. JP: Formal analysis, Writing – review & editing.
